# Yulu Shequ - a unique rehabilitation program for illicit drug users in Kaiyuan in southwest China

**DOI:** 10.1186/1477-7517-8-26

**Published:** 2011-09-20

**Authors:** Qinqin Liu, Christian A Gericke

**Affiliations:** 1 Faculty of Medicine, Dentistry and Health Sciences, University of Melbourne, Melbourne, Australia; 2 Professor of Public Health and Deputy Director, Peninsula CLAHRC, National Institute for Health Research, Peninsula College of Medicine and Dentistry, Universities of Exeter and Plymouth, Plymouth, UK

## Abstract

**Introduction:**

In China, illicit drug use and addiction have been rapidly increasing over the last two decades. Traditional compulsory rehabilitation models in China are widely considered ineffective. Recently, a new model of drug user rehabilitation called the 'Yulu Shequ Program' has gained a national reputation for successful rehabilitation in the city of Kaiyuan in southwest China. The aim of this study was to describe this program to the international public and to assess the program's effectiveness in terms of relapse rates and costs to participants and public payers.

**Case description:**

The Yulu Shequ program provides up to one hundred participants at any point in time with the opportunity to live and work in a purpose-built, drug-free community after completing compulsory rehabilitation. The length of stay is not limited. Community members receive medical and psychological treatment and have the option to participate in social activities and highly valued job skills training. The program has very strict policies to prevent illicit drugs entering the community.

**Evaluation:**

The evaluation was carried out through 1) a review of literature, official documents and websites in Chinese language describing the program and 2) an on-site visit and conduct of semi-structured interviews with key staff members of the Yulu Shequ program. The relapse rate in 2007 was 60% compared to 96% in the compulsory program. Annual costs to public payers of CNY4800 (US$700) were largely offset by income earned through on-site labour by participants totalling CNY4600 (US$670).

**Conclusions:**

The Yulu Shequ program is an interesting model for drug rehabilitation that could lead the way for a new Chinese national policy away from compulsory rehabilitation towards a more collaborative and effective approach. Caution is needed when interpreting relapse rates as Yulu Shequ participants need to have completed compulsory rehabilitation before entering the program. A more comprehensive evaluation of this program would be desirable before implementation in other parts of China or in other countries facing similar problems.

## Introduction

The number of drug users in China has rapidly increased as the availability of illicit drugs has become more widespread over the last twenty years. By the end of 2005, over one million people were registered drug users [1] representing just the tip of the iceberg of illicit drug abuse in China with estimates of the actual number of drug users totalling 3.5 million [2]. Injection drug use (IDU) contributes to a large share of fatal diseases including HIV/AIDS and hepatitis C. About half of all registered drug users in China inject drugs and about 42% of reported HIV/AIDS cases in China are attributable to IDU [3].

The city of Kaiyuan is located in the southwest region of China. It is near the 'Golden Triangle', including Myanmar, Laos, Vietnam and Thailand, where illicit drugs are produced in large quantities and the local government in Kaiyuan regards the reduction and prevention of illicit drug use as an important mission.

Traditionally, Chinese policy-makers have put great emphasis on supply reduction and abstinence therapy to control illicit drug use [4]. According to Chinese legislation, drug trafficking and abuse are illegal, and people who participate in drug trafficking can be severely punished [3]. The "Regulations on Prohibition against Narcotics" outline three levels of available treatment for drug users: 1) voluntary detoxification institutions run by the Department of Health; 2) compulsory detoxification institutions run by the Department of Public Security; and 3) "rehabilitation through-labour" units run by the Department of Justice [3,5,6]. In theory, convicted drug addicts are able to choose the rehabilitation option that best suits their situation. However, there are problems with the system. Most significantly, relapse rates are very high across all three rehabilitation pathways, and many "voluntary" patients cannot afford to go to a voluntary rehabilitation institution because they have to pay for it out-of-pocket. The average cost of attending voluntary rehabilitation is about CNY2000-5000 (about US$300-750). Therefore in practice, the compulsory detoxification or the rehabilitation-through-labour programs are the dominant pathways of rehabilitation for most drug addicts.

In Kaiyuan, local public security brought attention to issues with the traditional rehabilitation model and implemented a pilot program called 'Yulu Shequ' at an existing compulsory detoxification institution run by the Department of Public Security. The program was developed with the understanding that most addicts do not live in healthy social and personal environments due to a lack of acceptance by mainstream society and sometimes a lack of family support. The pilot program aims to reduce relapse rates by providing a healthy social environment which will ultimately foster reintegration into mainstream society. Recently, this pilot program has gained a national reputation for successful rehabilitation and could be the seed for a new era in Chinese drug offender rehabilitation using a gentler approach that could be a stepping stone towards an integrated harm reduction approach within the overall Chinese detoxification treatment policy which is still largely focused on a "zero tolerance" approach [6]. In fact, a number of harm reduction strategies including methadone maintenance treatment and needle exchange programs have been implemented over the last decade by the Ministry of Health [6]. However, these are mainly aimed at reducing the spread of HIV/AIDS and are not intersectorally integrated with the detoxification and rehabilitation programs run by the Departments of Justice and Public Security.

The aim of this study was to describe this pilot program to the international public and to assess the program's effectiveness in terms of reducing illicit drug abuse relapse rates and costs to participants and public payers.

To this end, we conducted 14 semi-structured interviews with key staff members of the Yulu Shequ program in Kaiyuan between January and March 2008, after permission for this study had been obtained from the Department of Public Security in Yunnan and from the Director of the Yulu Shequ program. The latter also nominated the staff members to be interviewed. Staff members included the head of the compulsory detoxification institution, supervisors, and nurses. The semi-structured interviews covered the following areas: 1) data on the infrastructure and on processes used in the program, 2) data on relapse rates and the definitions used for defining a relapse or a successful rehabilitation, 3) surveillance activities, 4) cost of rehabilitation to public payers and to addicts and their families, 5) data on successful reintegration into social life and employment, 6) health status data. Ethics approval (no.H-009-2008) for this study was obtained from the Human Research Ethics Committee at the University of Adelaide.

## Case description

The Yulu Shequ program has been set up as a drug-free community which consists of three components:

1) In the community there is a clinic which provides free health care for every participant including treatment for common drug associated diseases such as hepatitis. Monthly health checks are offered and the clinic is also responsible for random drug testing. In addition, dieticians regularly visit the community and prepare nutritious meals for residents.

2) Addicts are offered long-term psychological support in the community. A range of counselling sessions as well as sports and social activities are available for helping participants improve their social skills. For instance, there is a dance club and a basketball team to join. These help residents to develop friendships and explore other interests in life.

3) The third part of the program is the most unique and important. Several different processing factories operate on site and members have the opportunity to become involved in these companies, for example making jewellery. The companies supply all equipment and training courses for residents. A suitable position is offered to participants depending on their physical and psychological condition. Some of the jobs on offer include polishing glass into fake diamonds, electric welding and carpentry. The program ensures that every resident has the opportunity to learn certain skills through professional training and to have a paid job in the community. Ultimately, it aims to prepare addicts for life in wider society by rebuilding their self-confidence and self-esteem in order to adapt to normal social life.

There is no time limit for completing the Yulu Shequ program. Participants can live and work in the community as long as they want to and they can also withdraw at any time. The longest time a resident stayed in the community so far was 24 months. Furthermore, residents have some degree of freedom in the community, although they remain under supervision. They have the right to choose their roommates and to take holidays. They are able to leave the community to visit their families and friends after informing their supervisors. However, before they can re-enter the community routine drug tests are performed on every participant after an outside visit. This is flanked by a very strict policy to prevent illicit drugs from entering the community. The main types of drug tests used are urine and pupil tests which are used for testing cannabis, heroin, morphine and ice. Participants with a positive drug test are sent back to compulsory rehabilitation.

## Evaluation

### Relapse rates

Any participant completing compulsory rehabilitation in Kaiyuan is free to decide whether to leave rehabilitation or continue within the Yulu Shequ community. There are no further requirements or fees for entering the program. Since its inception in 2006, 555 people have participated in the Yulu Shequ program and 238 people were living the community in February 2008. Based on a retrospective analysis of routine institutional records by facility staff provided to the researchers, the illicit drug abuse relapse rate in the Yulu Shequ program in 2007 was 60% compared to 96% for the compulsory rehabilitation program run by the same institution. Because of the sequential order of the two programs and a selection bias due to the voluntary adherence to the Yulu Shequ program and the compulsory attendance of the basic rehabilitation program, no other comparator to evaluate relapse rates is available. Data on successful reintegration into social life and employment or on the health status of participants were not accessible.

### Costs

Annual average costs to public payers of CNY4800 (US$700) per program participant were largely offset by income earned through on-site labour by participants totalling CNY4600 (US$670). Approximately one third of costs were spent on the provision of medical care. Cost data were provided by the finance manager of the Kaiyuan Department of Public Security and could not be scrutinised independently.

## Conclusions

The Yulu Shequ program seems to achieve a far lower rate of relapse than the traditional, compulsory drug rehabilitation program alone. It needs to be emphasised that Yulu Shequ participants are a highly selected population as entry into the program is contingent on them having completed the standard compulsory detoxification program and participation is voluntary. Therefore direct comparisons of relapse rates with other first-line rehabilitation programs cannot be made. Because of its labour component the Yulu Shequ program appears to be largely cost-neutral to public payers. Possible additional benefits of reduced relapse rates include the reintegration of successfully rehabilitated addicts into society and a positive impact on drug-related crime. The Department of Public Security Yulu Shequ approach differs in many respects from the "rehabilitation through-labour" units run by the Department of Justice. In contrast to the latter, it is characterised by a more participative, gentler approach to drug addict rehabilitation, in many respects similar to therapeutic communities in western countries [7] with the main difference being a lack of integrated harm reduction strategies. The Department of Justice "rehabilitation-through-labour" units are considered incarceration sites, where addicts usually spend 2 to 3 years or "reeducation" without the permission to leave [6] whereas in the Yulu Shequ program participants are free to leave the community subject to drug use monitoring. The primary aims of the rehabilitation-through-labour units are to force drug users to quit drug use and to prevent them from committing crimes. In contrast to the Yulu Shequ program they do not emphasise health education, skills training [4] or social activities. Because of these characteristics the Yulu Shequ program is a rehabilitation program in the proper sense where participants are enabled to reintegrate into society once they leave the program whereas the "rehabilitation-through-labour" units have a markedly punitive character and have been called "labour camps" by other auhors [6]. Further study is required to undertake a more detailed evaluation of the program from the perspective of addicts, to provide a comparison of their experiences between the Yulu Shequ program and the traditional compulsory and voluntary rehabilitation programs. However, there is strong demand for places in the program which speaks for the popularity of the Yulu Shequ program among participants. Results from this further study may contribute to improvements of the rehabilitation system in other parts of China and in other countries with similar problems.

## Authors' contributions

QL and CAG planned the study. QL collected, analysed and synthesized the data; and wrote the first draft of the article. CAG assisted in data analysis and synthesis, and contributed to the writing of the article. All authors read and approved the final manuscript.

## Declaration of competing interests

The authors declare that they have no competing interests.
